# Supplementary figures and images for: Characterization of Transgenic Lines Labeling Reticulospinal Neurons in Larval Zebrafish
Source: eNeuro. 2025 May 27;12(5):ENEURO.0581-24.2025. doi: 10.1523/ENEURO.0581-24.2025 (PMC12119039; doi:10.1523/ENEURO.0581-24.2025)

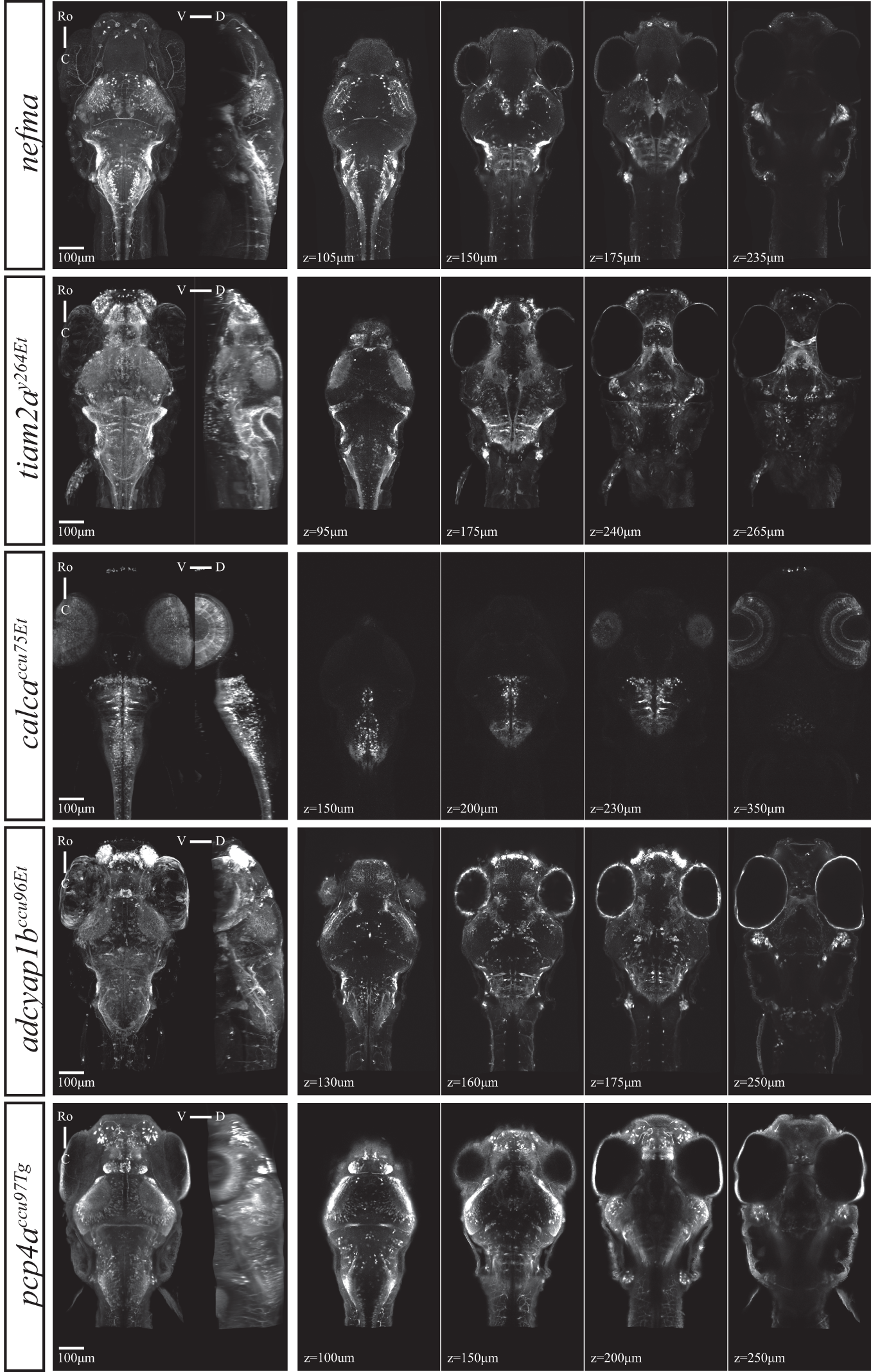

Supplement: Figure 1-1 — Example fish to show brain-wide expression patterns in selected transgenic lines. The nefma line has expression in the tectum, pre-tectum, tegmentum and hindbrain, as well as labelling the anterior and posterior lateral line ganglia, the trigeminal ganglion and neuromasts. The tiam2ay264Et line has expression in the olfactory epithelium, optic chiasm, tectum, interpeduncular nucleus, tegmentum, hindbrain and cerebellum. The calcaccu75Et line mostly labels cells in the hindbrain, as well as a small number of cells in the mid- and forebrain, and outer retina. The adcyap1bccu96Et line has expression in the olfactory epithelium, olfactory bulb, tegmentum, hindbrain, anterior and posterior lateral line ganglia, as well as sparse labelling in the tectum. The pcp4accu97Tg line has sparse labelling of cells in the forebrain and tectum, expression in the habenula, hindbrain, and the anterior and posterior lateral line ganglia. Scale bar is 100μm. Download Figure 1-1, TIF file. [file eneuro-12-ENEURO.0581-24.2025-s003.tif]

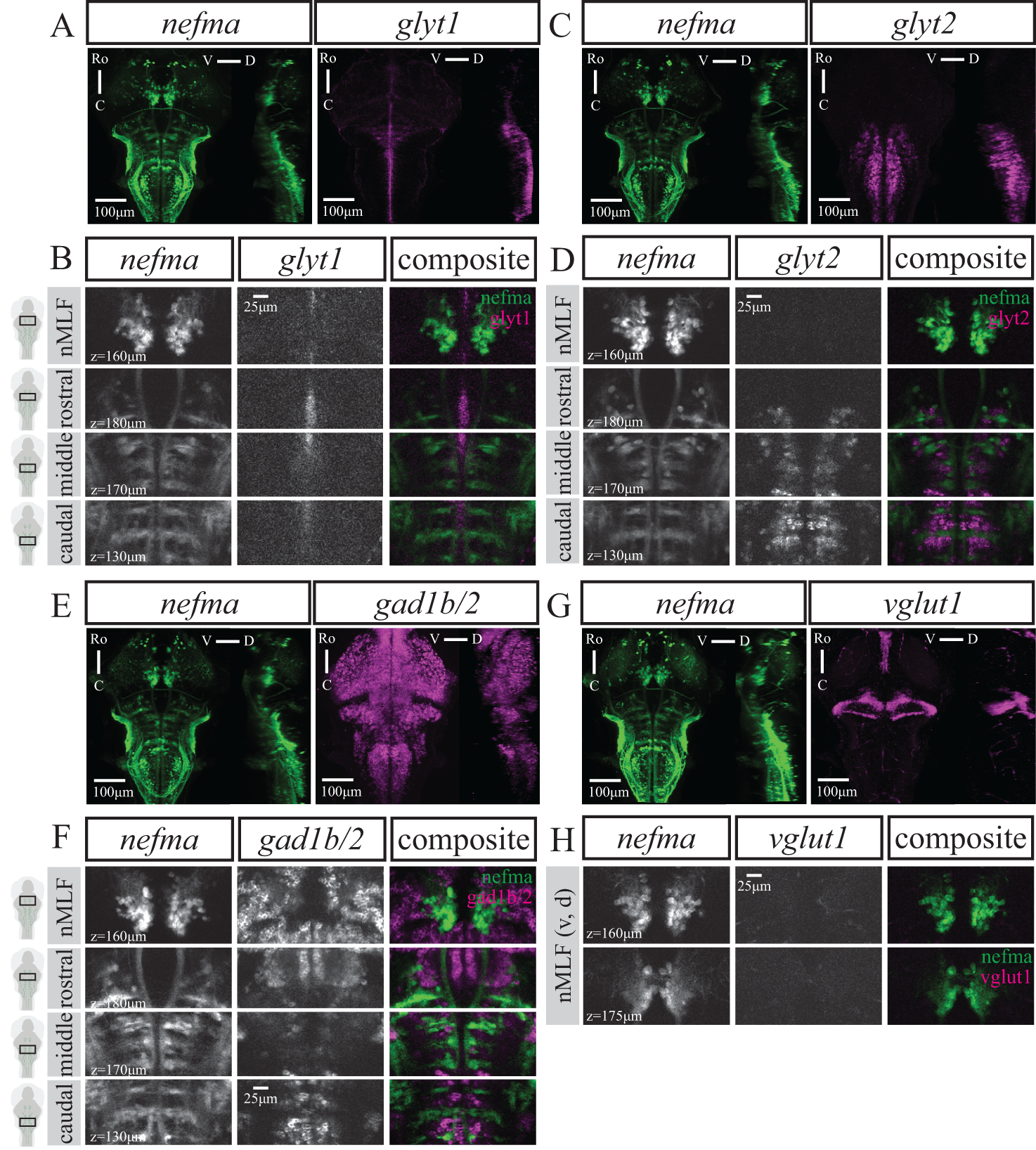

Supplement: Figure 2-1 — No GABAergic (gad1b/2), glycinergic (glyt1, glyt2) or glutamatergic (vglut1) expression in neurons labelled by the nefma line. A,C,E,G) Maximum intensity projections from dorsal and sagittal view of an exemplary nefma fish at 6dpf with A) gad1b/2 (n = 8), C) glyt1 (n = 16), E) glyt2 (n = 15) or G) vglut1 (n = 14) mRNA expression. Scale bar is 100μm. Close ups at several planes to better illustrate no overlap between nefma line and B) gad1b/2, D) glyt1, F) glyt2 or H) vglut1 mRNA-expressing neurons. Scale bar is 25μm. Download Figure 2-1, TIF file. [file eneuro-12-ENEURO.0581-24.2025-s004.tif]

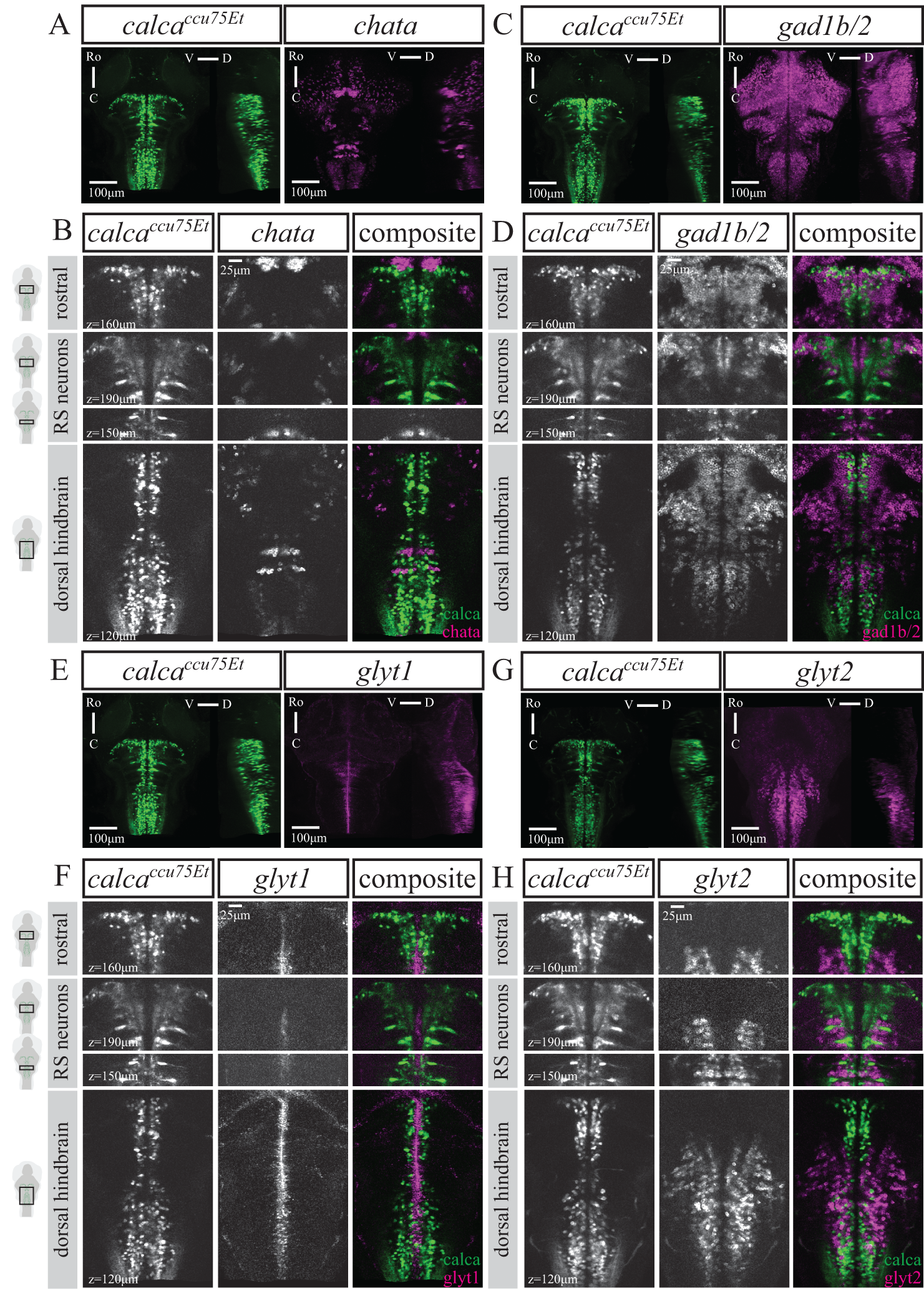

Supplement: Figure 3-1 — No cholinergic (chata), GABAergic (gad1b/2) or glycinergic (glyt1, glyt2) expression in neurons labelled by the calcaccu75Et line. A,C,E,G) Maximum intensity projections from dorsal and sagittal view of an exemplary calcaccu75Et fish at 6dpf with A) chata (n = 15), C) gad1b/2 (n = 7), E) glyt1 (n = 16), G) glyt2 (n = 16) mRNA expression. Scale bar is 100μm. B,D,F,H) Close ups at several planes to better illustrate no overlap between calcaccu75Et line and B) chata, D) gad1b/2, F) glyt1 or H) glyt2 or mRNA-expressing neurons. Scale bar is 25μm. Download Figure 3-1, TIF file. [file eneuro-12-ENEURO.0581-24.2025-s005.tif]

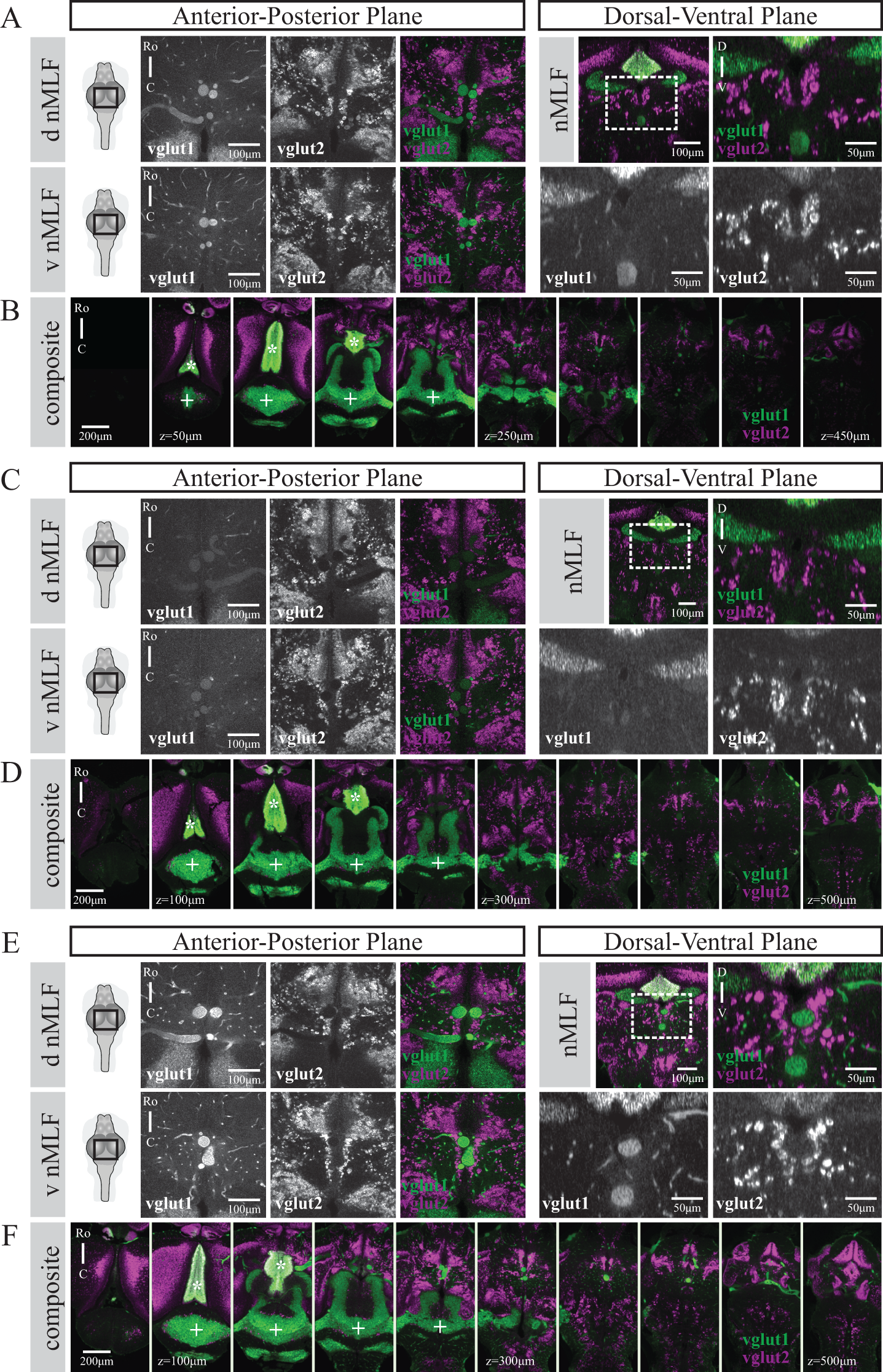

Supplement: Figure 5-1 — Glutamatergic expression patterns in three 4 week-old juvenile fish (of n = 4). A,C,E) Left panels show vglut1, vglut2, composite in two planes from the dorsal view, scale bar is 100μm. Right panels show vglut1, vglut2, composite from the transverse view, scale bar is 50μm. B,D,F) Composite images of several planes from dorsal to ventral. Note the presence of torus longitudinalis (*) and cerebellum (+) in vglut1 (green), with brain wide expression in vglut2 (magenta). Auto-fluorescence of blood vessels is seen in the vglut1 channel, as indicated by blue triangles. Scale bar 200μm. Download Figure 5-1, TIF file. [file eneuro-12-ENEURO.0581-24.2025-s006.tif]
